# Influence of Conspecific Male Odour and Host Kairomones on the Behaviour of *Sternochetus mangiferae*, a Pest of Mangoes in Brazil and Ghana

**DOI:** 10.1007/s10886-026-01696-5

**Published:** 2026-03-17

**Authors:** Miguel Borges, Mirian F. F. Michereff, Raúl A. Laumann, József Vuts, Marla J. Hassemer, João Victor C Machado, Michely F. S. Aquino, Kamila K S Leite, Alessandra de Carvalho Silva, Marcelo P Ricalde, Haruna Braimah, Michael A. Birkett, Maria Carolina Blassioli-Moraes

**Affiliations:** 1 Embrapa Genetic Resources and Biotechnology, Laboratory of Semiochemicals, Brasilia, DF Brazil; 2 Protecting Crops and the Environment, Rothamsted Research, Harpenden, Herts AL5 2JQ UK; 3 Embrapa Agrobiologia, Seropédica, RJ Brazil; 4 Institute Federal de Brasilia, Gama, DF Brazil; 5 CSIR-Crops Research Institute, Kumasi, Ghana

**Keywords:** Plant volatiles, Pheromone, Behavioural response, Mango weevil

## Abstract

*Sternochetus mangiferae* is a pest of mangoes, occurring worldwide in regions where mangoes are cultivated. In Brazil, this insect was first detected in 2014 in Seropédica, Rio de Janeiro, and since then, various measures and studies have been conducted to contain, monitor and potentially eradicate it. Semiochemicals may provide feasible targets to develop sensitive detection and monitoring tools. Therefore, this study aimed to evaluate whether *S. mangiferae* use pheromones from conspecifics and kairomones from mango tree host in their chemical communication. The findings identify a blend of mango volatiles that influence the behaviour of *S. mangiferae* females. A synthetic blend containing the EAD-active compounds γ-butyrolactone, benzaldehyde, 6-methyl-5-hepten-2-one, myrcene, (*Z*)-ocimene, 2-methoxyphenol, (*S*)-linalool, methyl salicylate and methyl benzoate was attractive to females of *S. mangiferae* from Ghanaian and Brazilian populations in two-choice olfactometry experiments. The results also indicate that the absolute configuration of linalool in the mango synthetic blend is crucial for female attraction, as females respond only to blends containing (*S*)-linalool. Furthermore, females from both populations were attracted to the odour of live males from their respective localities. Chemical analysis of aeration samples from the Brazilian population revealed that males and females emitted qualitatively similar volatile profiles. Although no male-specific compounds could be identified to fully explain female attraction, this study provides the first evidence of intraspecific chemical communication in *S. mangiferae*. These findings advance our understanding of the chemical ecology of this pest and support the development of semiochemical-based monitoring and management strategies for mango orchards.

**Supplementary Information:**

The online version contains supplementary material available at 10.1007/s10886-026-01696-5.

## Introduction

The mango weevil, *Sternochetus mangiferae* (Fab.) (Coleoptera: Curculionidae), is a quarantine pest widely distributed in mango-producing areas of Africa and Asia (Balock and Kozuma 1964; Pena et al. 1998) and is also present in Brazil. According to the Ministry of Agriculture, Livestock, and Supply of Brazil (MAPA), the insect is currently present in the state of Rio de Janeiro, specifically in the Metropolitan Region of Rio de Janeiro, and in the municipality of Oiapoque, Amapá state (2024). In the Rio de Janeiro region, there are few commercial mango production areas, so the impact of *S. mangiferae* in the country is still limited. However, there is concern about its potential expansion into commercial production areas in the states of São Paulo, Pernambuco, Bahia, Rio Grande do Norte and Ceará (Embrapa 2022). *Sternochetus mangiferae* is a monophagous insect, with adults emerging during the fruiting period, which in Rio de Janeiro occurs from September to February. Balock and Kozuma (1964) reported a diapause period of *S. mangiferae* in Hawaii that coincides with the non-fruiting period, indicating that the species’ biological cycle is synchronized with mango trees. In Brazil, there are no studies confirming diapause in local populations. However, given that *S. mangiferae* is a univoltine and monophagous species, it is reasonable to infer that the Brazilian population also has its biological cycle synchronized with mango phenology. *Sternochetus mangiferae* females lay eggs on mango fruits (Hansen et al. 1989). After hatching, the larvae penetrate the fruit pulp and feed while tunnelling toward the seed, where they complete most of their development until reaching the adult stage. Adults later exit the fruit to mate and initiate a new reproductive cycle. Because the insect spends most of its life cycle inside the fruit, infestations are difficult to detect. Damage caused by *S. mangiferae* can result in yield losses ranging from 5% to 90%, as infested fruits are unsuitable for export markets (Bagle and Prasad 1985; Verghese et al. 2000). The presence of the weevil in the fruit also creates quality control issues for the industry, as each fruit must be physically inspected before being processed. The pest status of *S. mangiferae* is based on four consequences of the infestation: (i) its development inside the fruit makes it non-marketable or less attractive; (ii) the mango pulp is damaged when the adult weevil emerges; (iii) weevil infestation reduces seed germination capacity, thus reducing the availability of seeds as a source of rootstock for propagation; and (iv) infestation causes premature fruit drop and poor fruit set (Follett 2002; Verghese et al. 2000).

In Brazil, *S. mangiferae* is under quarantine in the previously mentioned regions of Rio de Janeiro, and MAPA, together with Embrapa, has been monitoring the insect in the region. For these monitoring efforts, fruits must be collected from the area where the pest is present and then cut in half to detect the presence of larvae, pupae or adults inside (Silva et al. 2022). This procedure, given the number of plants and fruits that need to be sampled, is complex both in terms of logistics and the time required for fruit inspection. Therefore, semiochemicals-based tools, such as traps baited with kairomones or aggregations pheromones, offer a practical way to monitor *S. mangiferae* over long term and across wider areas, including for early detection in pest-free areas. Although the Curculionidae represent the most diverse animal family with more than 83,000 described species (Biedermann and Nuotclà 2020), semiochemicals have been identified for only 53 genera (El-Sayed 2025), including aggregation pheromones for 49 species across 25 genera (El Sayed 2025; Bandeira et al. 2021). In general, curculionid pheromones are terpenoid derivatives and multicomponent mixtures, such as in the genera *Anthonomus* and *Pissodes*, which produce grandlure I, II, III and IV as the main components of their aggregation pheromone. The genera *Rhynchophorus*, *Metamasius*, *Rhabdoscelus*, *Scyphophorus* and *Sphenophorus* produce alcohols or ketones as aggregation pheromones (Bartelt 1999; Bandeira et al. 2021). In addition to pheromones, the synergistic interaction of plant volatiles and pheromones has been demonstrated in Y-tube olfactometer bioassays and field experiments. The cotton boll weevil, *Anthonomus grandis* Boheman, is attracted to six volatile compounds emitted by cotton plants at the reproductive stage, whose activity is synergistically enhanced for both sexes by the aggregation pheromone in Y-tube olfactometer assays (Magalhães et al. 2016). Males of the red palm weevil, *Rhynchophorus ferrugineus* Olivier, produce 4-methyl-5-nonanol and 4-methyl-5-nonanone as aggregation pheromone, whose attractiveness is synergised by palm bait volatiles, each otherwise only weakly attractive alone (Giblin-Davis et al. 1996; Guarino et al. 2011; Vacas et al. 2017). Guarino et al. (2011) demonstrated significantly higher captures of *R. ferrugineus* when pheromone traps were combined with two palm esters, ethyl propionate and ethyl acetate, compared with traps baited with the pheromone alone or with only one of these esters. Similarly, the capture of *Conotrachelus nenuphar* Herbst, an important pest of plum orchards, in pheromone traps is enhanced by combining the plant volatile benzaldehyde with its aggregation pheromone (grandisoic acid) in a ratio of 10:1, respectively (Piñero and Prokopy 2003). Although there are studies reporting on the biology and ecology of *S. mangiferae*, information on its reproductive behaviour and intraspecific communication is scarce. Recently, a study reported that both males and females use stridulatory signals during reproductive behaviour (Catafesta et al. 2023). However, there is still no information on whether mango kairomones or pheromones influence the behaviour of *S. mangiferae*. Therefore, in this study, we evaluated whether kairomones emitted by mango trees affect the behavioural responses of *S. mangiferae* and whether pheromones are used by males and females in intraspecific communication.

## Methodology

### Insects

*Sternochetus mangiferae* were collected from mango stones in orchards at Embrapa Agrobiology and UFRRJ, Seropédica (22°44′ 9″ S, 43°42′19″ W), Rio de Janeiro state. In Brazil, during the fruiting season the insects were collected from mango stone following the methodology reported in Kamala Jayanthi et al. (2012). During the off-season, insects were collected by shaking trees over a white cloth, and by actively searching under the bark and among epiphytes and moss on stems and branches. These active searching procedures were also commonly used to collect insects in Ghana. The mango stone found in the ground around the trees were brought to the laboratory and maintained in plastic boxes with humified vermiculite in a climatized room (25 ± 2 °C, 60% relative humidity, and a 12:12 h photoperiod). The boxes were monitored daily, and any insects found were sexed and transferred to separate plastic boxes, one for males and another for females, where they were maintained with small pieces of mango fruit. Insects collected in the field by active monitoring were maintained in the same way. The insects were transported to Embrapa Genetic Resources and Biotechnology, Brasília, where they were kept in quarantine according to regulations established by the Ministry of Agriculture (MAPA - Transport and Research Authorization Document 183/2020/DSV/DAS/MPA) for subsequent aeration and behavioural studies. The insects were kept in acrylic boxes with perforated lids (Gerbox, 11 × 11 × 3.5 cm) in groups of 25 to 30 (males or females). The insects were sexed based on sexual dimorphism described by Unahawutti (2015). The boxes were sealed with rubber bands and placed inside a plastic container with a lockable lid, properly ventilated. The insects were fed a natural diet using small pieces of mango, which were replaced every two days. For electroantennography and some behavioural studies with volatiles from mango trees, insects were collected from orchards at the CSIR-Crops Research Institute Kwadaso Station (Kumasi, Ghana) and maintained at Rothamsted Research (England) using the procedures described above. In both Brazil and England, the insects were kept in quarantine rooms under controlled climate conditions at 25 ± 2 °C, 60% relative humidity, and a 12:12 h photoperiod. The age and mating status of the insects used in EAG experiments and behavioural assays conducted in the UK were unknown. In Brazil, the insects used in behavioural assays and volatile collections were separated by sex immediately after emerging from the mango stones, allowing us to infer that most individuals were unmated. However, their age was not controlled, and the insects used ranged from 15 to 90 days after collection from the mango stones. Mango volatiles previously evaluated with the Ghanaian *S. mangiferae* population were tested with a Brazilian population from Seropédica, Rio de Janeiro. Additionally, studies to identify pheromones were also carried out in Brazil.

## Collection of Volatiles

The collection of volatiles produced by *S. mangiferae* males and females and from mango fruits and inflorescences was conducted using the aeration technique with the insects from Seropédica (RJ) Brazil. Volatile collections from insects were conducted in two independent experimental sets using different groups of insects. In the first experiment, thirty-five males and thirty-five females of unknown age were placed separately in 700 mL horizontal glass chambers. Volatiles were collected over two consecutive days, with sampling performed every 24 h. After each collection period, a new set of insects was used. This procedure resulted in a total of five biological replicates, yielding 10 volatile samples from males and 10 volatile samples from females. In the second experiment, 94 females and 128 males were placed separately in the same type of glass chamber described above. Volatiles were collected over six consecutive days, with one biological replicate per sex every 24 h. The air inlet of each chamber was connected via silicone tubing to a water bubbler fitted with an activated charcoal filter (20–40 mesh; Supelco, Bellefonte, PA, USA) to humidify and purify the incoming air. The air outlet of the chamber lid was connected to a glass tube containing 100 mg of Porapak Q adsorbent (60–80 mesh; Supelco) for trapping headspace volatiles. The adsorbent tube was connected at the opposite end, via silicone tubing, to a vacuum pump, establishing a continuous airflow through the chamber at 0.6 L min⁻¹. After collection, the adsorbent was eluted with 0.5 mL of distilled hexane to recover the trapped volatiles.

Volatiles from mango fruit (var. Coquinho) and inflorescence (var. Tommy) were also collected (*n* = 5) in Brazil. The size of the inflorescences was uniformized, measuring approximately 30 cm, whereas the fruit measured 2.5 to 3.0 cm, with mean ± standard deviation weight of 6.61 ± 1.63 g, as insects prefer to bore into young fruits of this size (Braimah and van Emden 2010). The fruits and inflorescence were placed in nylon plastic bags (482 × 596 mm, Oven Bags, Reynolds, Lake Forest, IL, USA), with the opening sealed with plastic-coated wire tape (as described in Borges et al. 2020). Before use, the oven bags were conditioned and cleaned in an oven for 40 min at 100 °C, after being turned inside out. Air entered the plastic bags through a compressor with an airflow of 1 L·min⁻¹, connected to an activated charcoal filter (20–40 mesh, Supelco, Bellefonte, PA, USA), allowing only purified air to enter. The air was withdrawn through a vacuum pump with a flow rate of 0.6 L·min⁻¹, connected to a glass tube (10 cm length, 0.5 cm ID) containing 100 mg Porapak Q (60–80 mesh, Supelco, Bellefonte, PA, USA). All connections were made using polytetrafluoroethylene (PTFE) tubing, creating a positive pressure system. Volatiles were collected for 24 h and eluted from the adsorbent tubes using 0.5 mL hexane. The obtained samples were stored at -20 °C until they were used for chemical analyses or behavioural bioassay. An internal standard, *n*-heneicosane, with a final concentration of 0.01 mg/mL, was added (1 µL) to the sample to quantify the compounds via the internal standard method. The response factor for the compounds was assumed to be 1. All samples were then concentrated to 50 µL under a nitrogen stream.

## Chemical Analysis

For quantitative analysis, the volatile samples from the aeration of males and females, fruit and inflorescence were analysed using an Agilent 7890B gas chromatograph equipped with a split-splitless injector, a flame ionization detector (FID) and a non-polar DB-5MS column (30 m × 0.25 mm ID, 0.25 μm film thickness, J & W Scientific). The oven temperature for all samples was maintained at 40 °C for 1 min, programmed to increase at 5 °C/min to 150 °C, held for 0.1 min, and then increased at 10 °C/min to 250 °C. Two µL of each sample were injected on GC. Helium was used as the carrier gas. For qualitative analysis, selected aeration samples were analysed by gas chromatography-mass spectrometry (GC-MS) (Agilent 5975-MSD) equipped with a quadrupole analyser, using a non-polar DB-5MS column (0.25 mm diameter x 30 m length, with a 0.25 μm film thickness, Supelco, Bellefonte, PA, USA), in splitless mode, with helium as the carrier gas. Two µL of each sample were injected. The same temperature program used for GC analysis was followed. Data were collected and analysed using the MassHunter Workstation 10.1.49 software (Agilent, USA). Compounds were preliminarily identified by comparing the fragmentation pattern of the mass spectra with the NIST 20 library database and calculating the retention index (RI) by comparing the retention times with a linear alkane series (C8-C26) run under the same conditions. For final confirmation, the fragmentation pattern and RI of the compounds were compared with authentic standards, when available.

## Absolute Configuration of Linalool Produced by Mango Trees

This analysis was conducted because linalool elicited an antennal response, and as demonstrated for other insect species, the absolute configuration of plant-produced compounds can strongly influence insect behavioural and physiological responses (Mori 2014; Hassemer et al. 2016; Sims et al. 2022). The stereochemistry of linalool was determined using an Agilent 7890 A gas chromatograph equipped with a splitless injector and an FID, using a chiral β-cyclodextrin 360 capillary column (30 m × 0.25 mm ID, 0.25 μm film thickness, Supelco, USA). The GC oven was held at 40 °C for 1 min, programmed to increase at 5 °C/min to 210 °C, and held for 10 min. Helium was used as the carrier gas. A 2 µL aliquot containing both linalool enantiomers was injected into the chiral GC column to verify their successful separation, followed by co-injection with the inflorescence and fruit samples, (*R*)-linalool and also a co-injection of inflorescence with racemic mixture of linalool, co-injection of fruit sample volatile with racemic mixture of racemic linalool to check the absolute configuration of linalool produced by the mango tree.

## GC-EAD

Coupled GC-electroantennography analyses were performed as exploratory assays to identify volatile compounds potentially involved in the attraction of *S. mangiferae* to its mango host. The EAG-active volatiles identified through these exploratory recordings guided the selection of compounds subsequently evaluated in behavioural assays to confirm their roles in *S. mangiferae* behaviour. Coupled GC-electroantennography analyses were performed using stainless steel electrodes, with electrical connections made using conductive gel. One antenna of the female mango weevil was excised and mounted between the electrodes. Females were used for the EAD recordings, because they rely on olfactory cues to locate fruits for oviposition. The ends of the flagellum and scape were trimmed with a scalpel to ensure good electrical contact. Volatile extracts from mango inflorescences and fruits (Borges et al. 2020) were analysed using an Agilent 6890 N gas chromatograph equipped with a cold on-column injector, an FID and a non-polar DB-1 column (50 m × 0.32 mm ID, 0.52 μm film thickness, J & W Scientific). The GC oven temperature was maintained at 30 °C for 2 min and then programmed to rise at 5 °C/min to 100 °C and 10 °C/min to 250 °C. Helium was used as the carrier gas. Antennal responses were measured in mV deflections, and the signals were transmitted through a high-impedance amplifier (UN-06, Syntech, Netherlands). Simultaneous recordings of EAD and FID responses were analysed using a custom software package (EAD version 2.3, Syntech, Netherlands). Compounds eliciting an EAG response from at least three antennae at the same retention time were considered electrophysiologically active (Arx et al. 2011; Magalhães et al. 2018). To confirm compound identity for EAD-active peaks, their retention index was calculated using a linear saturated alkane series (C9-C24).

### Chemicals

Authentic chemical standards of γ-butyrolactone, 2-methoxyphenol, α-pinene (98%), ocimene (90%), linalool (98%), (*R*)-linalool (95%), racemic linalool (97%), β-myrcene (≥ 90%), 3-carene (≥ 90%), limonene (96%), (*E*)-ocimene (mixture of isomers 30% *Z*, 70% *E*), benzoic acid methyl ester (> 98.5%), benzoic acid ethyl ester (≥ 99%), methyl salicylate (≥ 98%) and geranylacetone (96%) were purchased from Sigma-Aldrich Inc (Sigma Aldrich, Steinheim, Germany). (*S*)-linalool was obtained by hydrodistillation from coriander seed oil, followed by purification on a silica gel column (Hassemer et al. 2016). (*E*)-4,8-Dimethyl-1,3,7-nonatriene (DMNT) (95%) and (*E-E*)-4,8,12-trimethyl-1,3,7,11-tridecatetraene (TMTT) (97%) were synthesized from geraniol and (*E*,* E*)-farnesol, respectively (Leopold 1986).

## Behavioural Bioassays with Linear Two-Choice Olfactometer

To evaluate the response of males and females to odour from conspecifics, bioassays using a linear two-choice olfactometer comprised of three chambers were used in England with insects from Ghana. The linear two-choice olfactometer had two circular acrylic side chambers with movable lids, where the odours to be tested were placed (Fig. S1). These chambers were connected to two activated charcoal filters (20–40 mesh, Supelco), through which filtered air entered. The central chamber, also with a removable lid, was the release area for the insects, connected to the side chambers by acrylic tubes. The external sides of the olfactometer were covered with black adhesive tape to limit the effect of visual cues for test insects. The central chamber was connected to a vacuum pump via a silicone tube, creating an airflow through the charcoal filters, side chambers, connecting tubes, and finally exiting the arena after passing through the central chamber (Fig. S1). The flow rate was 0.6 L/min. For each bioassay, insects were released into the central chamber and observed for 10 min, with the final chamber choice recorded. To evaluate the period of the activity of the insects, the bioassays were carried out during two periods: daytime from 9:00 to 17:00 and nighttime from 20:00 to 24:00. For bioassays with live insect odours, 10 males or 10 females were placed inside a small stainless-steel cage that was put into one of the side chambers. In the central chamber, another group of 10 males or females was released. Insect choices were recorded after 2 hours. The tested treatment combinations were: (i) odour of 10 live males vs. odour of 10 live females, (ii) odour of 10 live males vs. clean air, (iii) odour of 10 live females vs. clean air, and (iv) male aeration extract vs. diethyl ether. For the bioassays using male aeration extract, ten microliters of extract or diethyl ether were applied onto filter papers (1 cm^2^), which remained at room temperature for 1 min before being inserted into the chamber, thus allowing the solvent to evaporate. Each experiment was replicated 10 times, with each group of 10 insects used only once. For these bioassays, groups of insects were used, providing a clear behavioural response, whereas the responses of individuals were not consistent. We based the methodology on previous experience of our research group with the banana weevil, *Cosmopolites sordidus* (Abagale et al. 2019). In addition, testing insects in groups allows a higher number of individuals to be evaluated simultaneously and increases the robustness of the data. However, when using this approach, we acknowledge that volatile compounds released by the tested individuals may potentially influence insect responses. This limitation is considered and discussed in the Discussion section.

To evaluate the response of males and females to synthetic solutions of EAG-active compounds, 10 µL of the synthetic solution (Table 1) containing the compounds from fruits with racemic, as well as (*R*) and (*S*), linalool was evaluated. The aliquot was added to a filter paper, which was placed in one of the side chambers of the olfactometer, while a filter paper with 10 µL hexane as control was placed in the other side chamber. The filter papers were replaced after each bioassay. For each bioassay, an insect was released into the central area and observed for 10 min, with the final chamber choice recorded. The synthetic solutions containing mango volatiles were applied in similar amounts found in the volatile samples collected from mango fruits (Fig. S1). Based on these results, we proposed a synthetic mixture (Table 1). The number of replicates per treatment ranged from 49 to 53. In all bioassays, the olfactometers were cleaned after every five bioassays, and odour source positions were alternated to prevent side bias.

**Table 1:** Compounds with electroantennographic activity and their quantities used in the synthetic blends (10 µL) for evaluating insect responses in olfactometry bioassays

Compounds	Amount mg/mL fruit mix	
γ-Butyrolactone		0.005
Benzaldehyde		0.01
6-Methyl-5-hepten-2-one		0.01
Myrcene		0.5
(*Z*)-Ocimene		0.1
2-Methoxyphenol		0.03
(*R*)-Linalool / (*RS*)-Linalool		0.02
Methyl salicylate		0.1
Methyl benzoate		0.2
(*S*)-linalool		0.02

## Y-tube Olfactometer Bioassays

To assess whether insects from a Brazilian *S. mangiferae* population would exhibit similar behaviour to those from Ghana, Y-tube olfactometer bioassays were conducted in Brazil with insects from Seropédica, RJ. The olfactometer consisted of a square acrylic block (19 × 19 × 1.5 cm), with a Y-shaped cavity 1 cm wide situated between two glass plates (Moraes et al. 2008). The length of the main arm of the cavity was 12 cm, and each branch of the Y was 16 cm. Filter papers containing the volatiles to be tested were placed into 10 mL glass syringes connected to the olfactometer arms via silicon tubing. Charcoal-filtered and humidified air was pushed through the system at 0.6 L/min and pulled out at 0.6 L/min via a push-pull system. The insects (male or female *S. mangiferae*) were individually introduced at the base of the Y-tube and observed for 600 s. The first choice, defined as the first arm the insect entered and remained in for at least 30 s, was recorded. After every five repetitions, the positions of the arms of the olfactometer were alternated to avoid bias in the insect’s responses. As with the linear olfactometer, aliquots of the synthetic solutions were replaced after each bioassay. The insects’ responses were evaluated for the following treatments: (i) 10 µL of the synthetic solution containing the fruit compounds with (*S*)*-*linalool vs. hexane (Table 1); (ii) 10 µL male aeration extract vs. hexane; (iii) 10 live males vs. air; (iv) 10 live females vs. air; (v) 10 live males + synthetic solution containing the fruit compounds with (*S*)*-*linalool vs. air; (vi) and 10 live males + synthetic solution containing the fruit compounds with (*S*)*-*linalool vs. 10 live males. Ten microliters of each treatment or hexane control were applied onto filter papers (1 cm^2^), which remained at room temperature for 1 min before being inserted into the syringe, thus allowing the solvent to evaporate. The bioassays v) and vi) were performed only with females, since only females responded to male volatile extracts and live male odour. Treatments (solutions or live insects) were introduced in glass syringes placed before the air entrance in the olfactometer. The number of replicates for each treatment varied from 36 to 96 insects tested. The different numbers of insects tested among treatments were due to a high proportion of non-responsive individuals in some assays. Bioassays were therefore replicated until at least 30 insects had made a choice, ensuring sufficient data for analysis. These bioassays were conducted between 10:00 to 15:00 with insects maintained in an inverted photoperiod (referent to 19:00–24:00), using only a red bulb lamp (10 W, Luminatti, SP- Brazil) for illumination. The olfactometers were cleaned after every five bioassays, and odour source positions were alternated to prevent side bias.

### Statistical Analysis

First-choice data from the bioassays were analysed using the chi-square test in R software (version 4.11) (α = 0.05). The quantities of volatiles in male and female extracts were compared using the *t*-test (PAST, version 4.17 C).

## Results

### Chemical Analysis of Fruit and Inflorescence Volatile Samples

The chemical analysis of mango fruit (var. Coquinho) headspace samples showed the presence of 24 major compounds, with β-myrcene, 3-carene and ethyl and methyl benzoate released in higher levels compared to other volatile compounds. We identified 32 major compounds in the inflorescence volatile samples. Among them, the monoterpenes β-myrcene, 3-carene, (*Z*) and (*E*)-ocimene, α-terpinolene, the esters methyl and ethyl benzoate and the sesquiterpenes α-gurjunene and α-farnesene were released at higher levels (Table S1). 

### GC-EAD with *S. mangiferae* Antennae

**Fig. 1:**
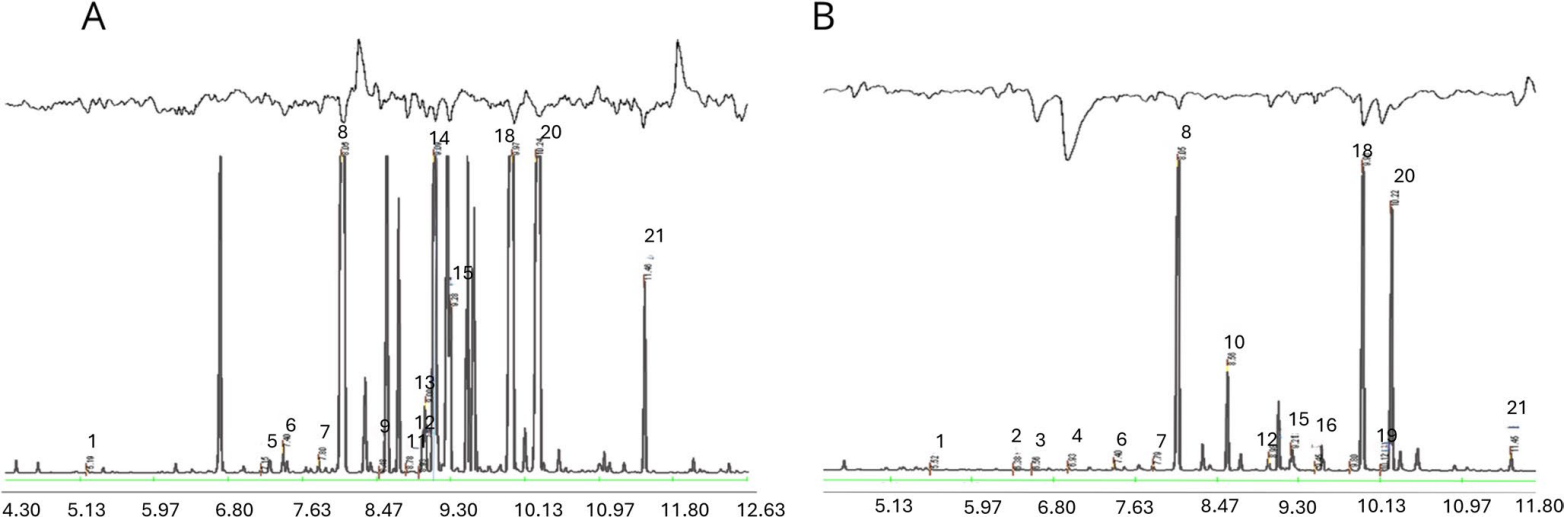
Female antennal responses in GC-EAD assays. **A** mango inflorescence volatiles, **B** mango fruit volatiles. Compound name/RI: (1) methyl-2-methyl butyrate/766, (2) γ-butyrolactone/860, (3) (*E*)-2-hexen-1-ol/873, (4) anisole/898, (5) unidentified/916, (6) benzaldehyde/937, (7) 6-methyl-5-hepten-2-one/967, (8) myrcene/987, (9) limonene/1023, 11) (*Z*)-ocimene /1049, 12) methyl benzoate/1062, 13) 2-methoxyphenol (guaiacol)/1067), 14) terpinolene /1075, 15) linalool/1083, 16) DMNT/1105, 18) ethyl benzoate/1153, 20) methyl salicylate/1178, 21) methyl-2-methoxybenzoate /1299. (RI on a DB-1 column)

Female *S. mangiferae* antennae showed responses to 14 peaks in inflorescence and fruit extracts, identified as: benzaldehyde, 6-methyl-5-hepten-2-one, myrcene, methyl benzoate, (*Z*)-ocimene, linalool, ethyl benzoate, methyl salicylate, methyl-2-methoxybenzoate, limonene, 2-methoxyphenol, γ-butyrolactone and (*E*)-2-hexen-1-ol, and one unidentified compound 5 (Figs. 1A and B).

### Behavioural Studies with Conspecific Odours

**Fig. 2:**
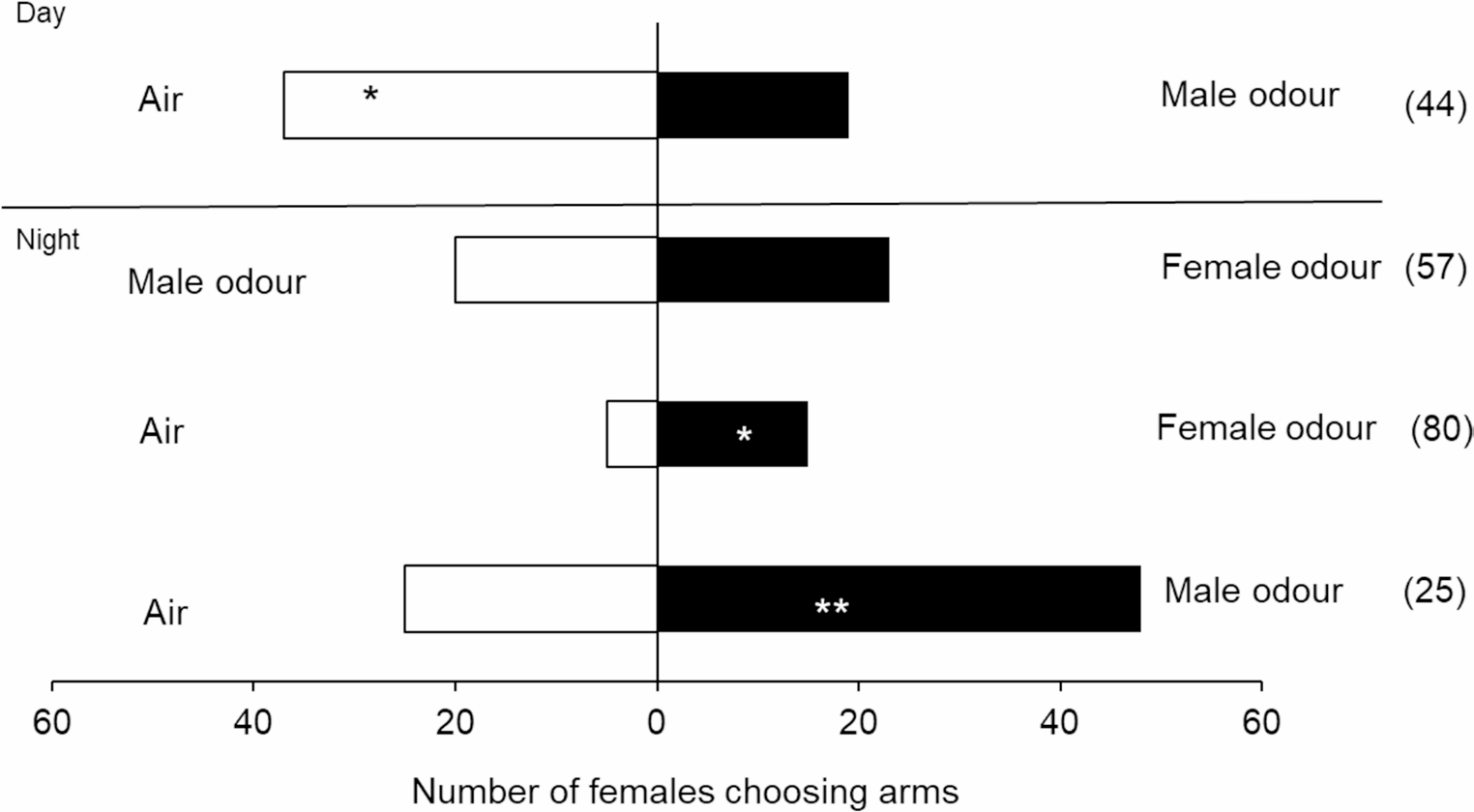
Behavioural response of *S. mangiferae* females from a Ghanaian population in the linear olfactometer to volatiles from conspecific males and females. The number in parentheses indicates non-responsive insects. * Significant differences between treatment and control (ꭓ^2^, *P* < 0.05)

**Fig. 3:**
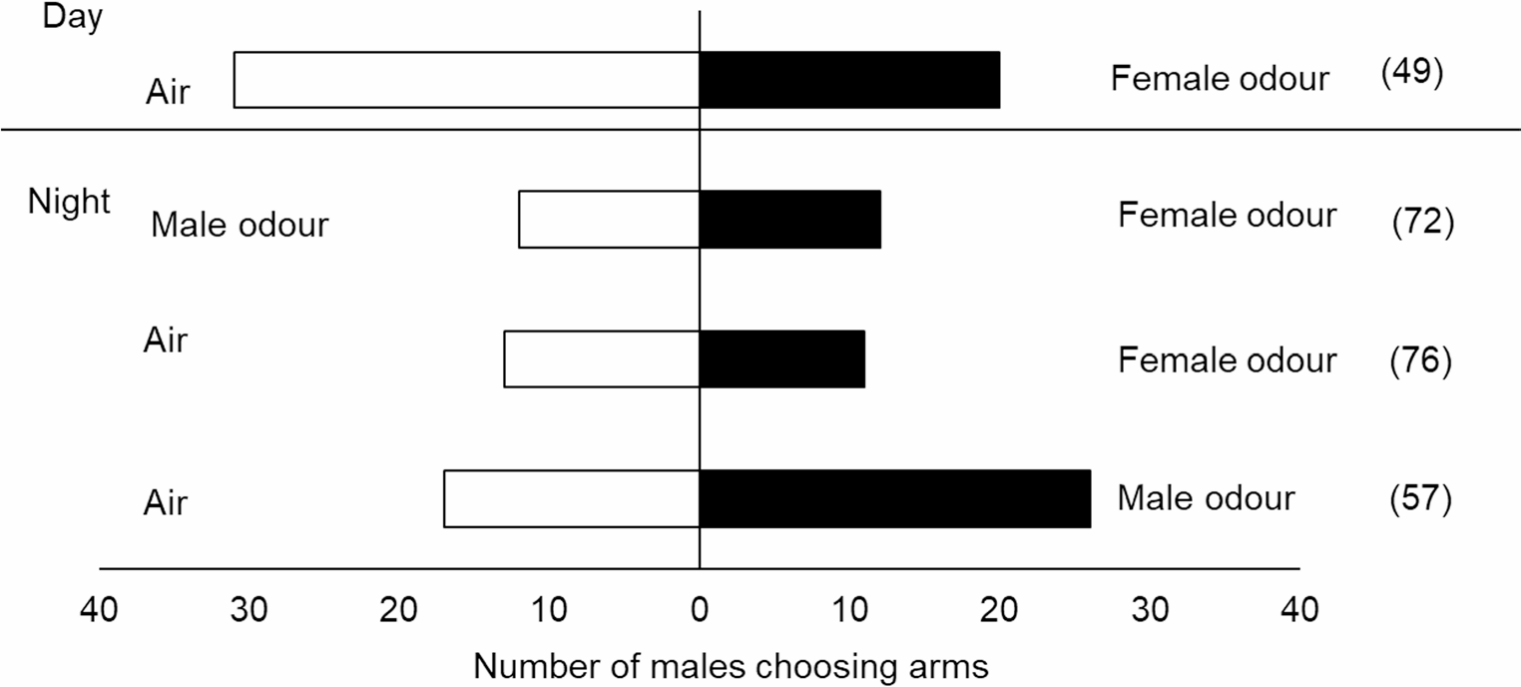
Behavioural response of *S. mangiferae* males from a Ghanaian population in the linear olfactometer to volatiles from conspecific males and females (ꭓ^2^, *P* < 0.05). The number in parentheses indicates non-responsive insects

No consistent responses to the odour of live insects were observed with individuals from the Ghana population, and there was a high number of non-responsive insects during the daytime; also, females preferred blank air during this period (χ² = 5.786, *P* = 0.016) (Fig. 2). However, in the bioassays conducted at night (20:00 to 24:00), females showed a preference for male odours (χ² = 7.246, *P* = 0.007). Despite the high number of non-responsive individuals (80%), females also showed a preference for female odours (χ² = 5.000, *P* = 0.025) and did not show a preference when male odours were contrasted with female odours (χ² = 0.209, *P* = 0.647) (Fig. 2; Table S2). When evaluating the response of males from the Ghanaian population to conspecific odours, no preference for either male or female odours was observed when contrasted with air in both periods evaluated (Fig. 3; Table S2). As individuals exhibited a higher response during the scotophase, all subsequent bioassays were conducted under scotophase conditions.

### Behavioural Responses to Synthetic Mango Blends

**Fig. 4:**
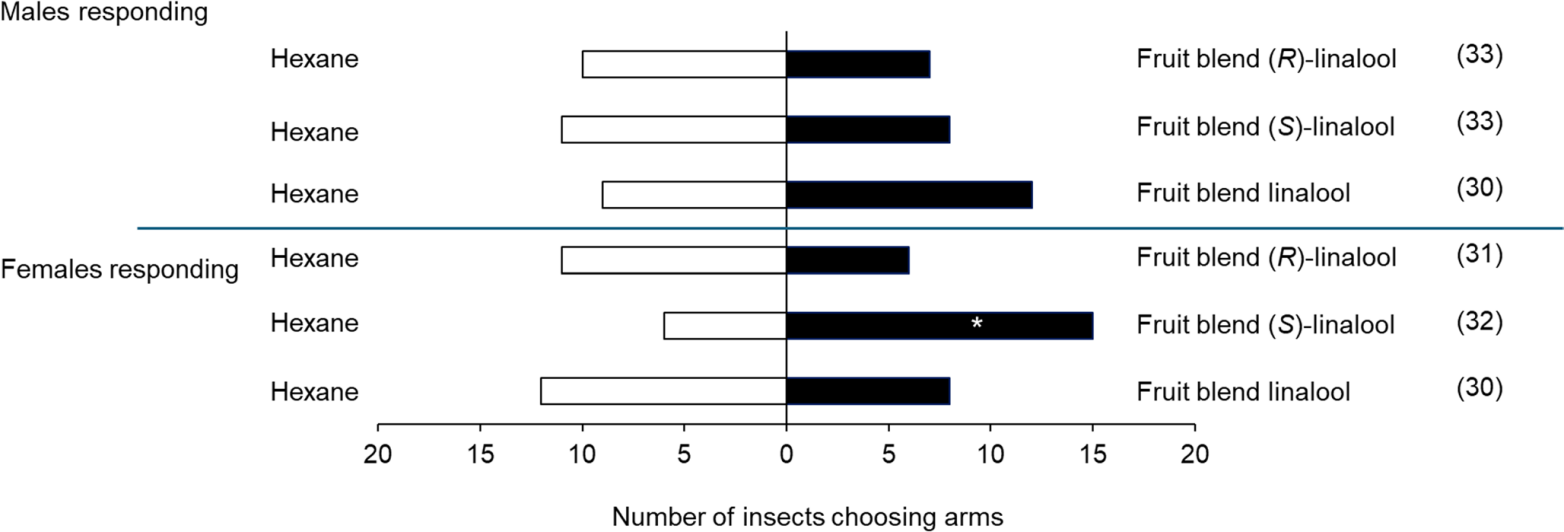
Behavioural response of *S. mangiferae* males and females from a Ghanaian population in a linear olfactometer to synthetic blends of volatiles. The number in parentheses indicates non-responsive insects. * Significant difference between treatment and control (ꭓ^2^, *P* < 0.05). Linalool = *rac*. linalool

When evaluating the response of *S. mangiferae* males and females from Ghana using synthetic mixtures with (*S*), (*R*) or racemic linalool, females showed a significant preference for the synthetic mixture with (*S*)-linalool, compared to hexane (χ² = 3.483, *P* = 0.05). Females did not show preference to all other treatments, while males showed no preference for any treatments (Fig. 4; Table S2).

**Fig. 5:**
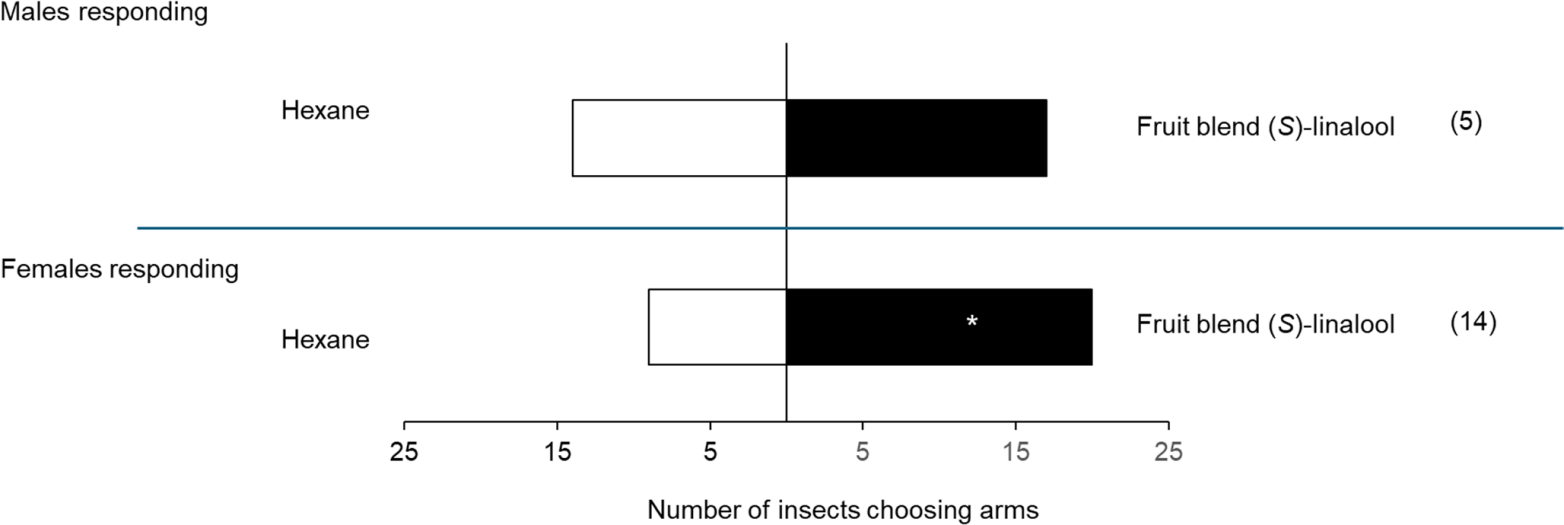
Behavioural response of *S. mangiferae* males and females from a Seropédica (RJ) population in a Y-tube olfactometer to a synthetic blend with (*S*)-linalool, compared to hexane. The number in parentheses indicates non-responsive insects.* Significant difference between treatment and control (ꭓ^2^, *P* < 0.05)

*Sternochetus mangiferae* females from a Seropédica (RJ) population also showed preference for the synthetic mixture with (*S*)-linalool in Y-tube olfactometer assays (χ² = 4.172, *P* = 0.04). The males did not show preference when contrasting the odour from synthetic mixture against hexane (Fig. 5; Table S2).

### Absolute Configuration of Linalool Present in Mango Fruits and Inflorescences

**Fig. 6:**
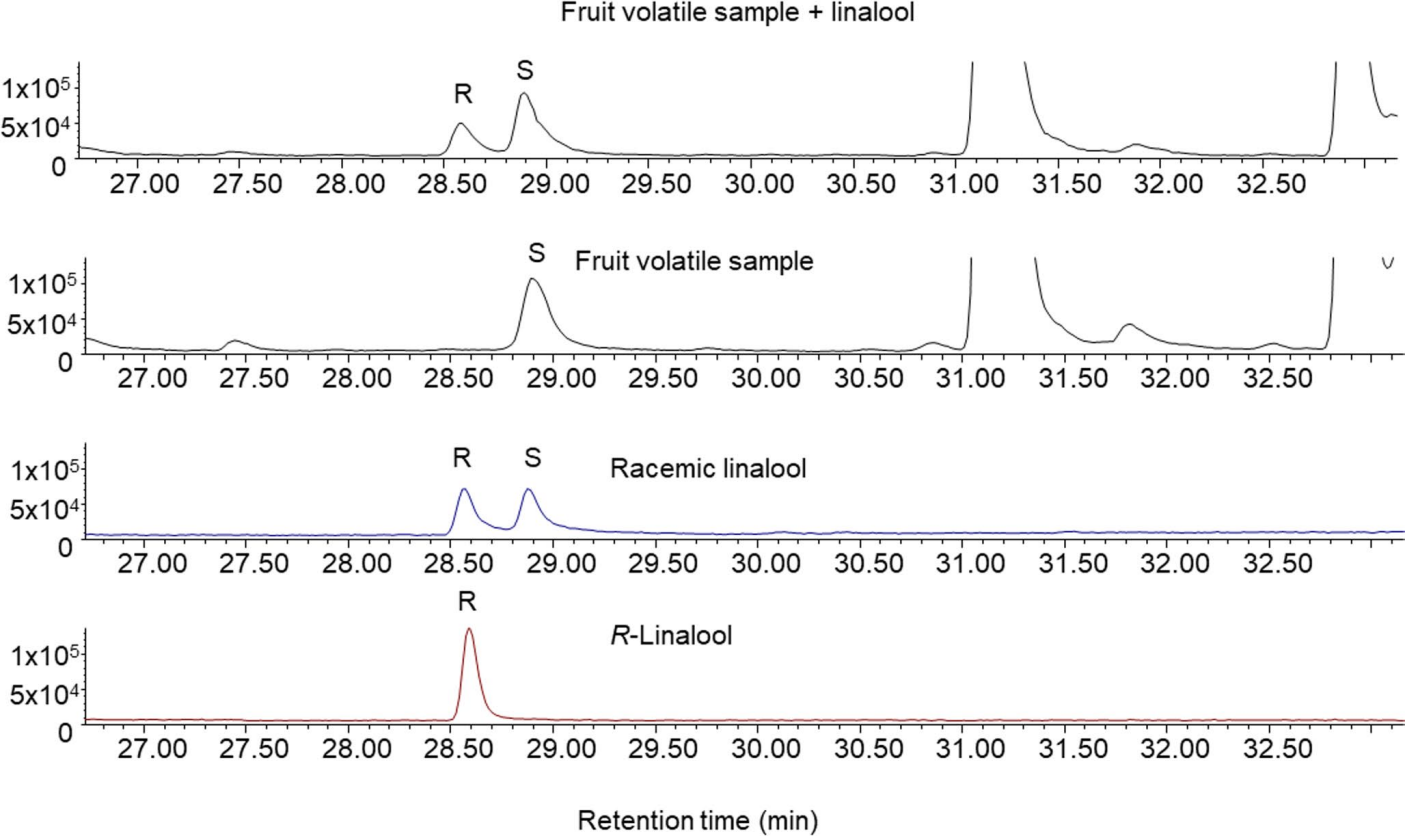
**A** GC co-injection of a mango fruit (var. Coquinho) aeration extract with racemic linalool, **B** fruit aeration extract, **C** synthetic racemic linalool, **D** synthetic (*R*)-linalool. Column: β- DEX-360

Since only the mixture containing (*S*)-linalool elicited a behavioural response, chiral GC analysis was conducted on the inflorescence and fruit extracts to verify whether the linalool isomer produced by mango inflorescence and fruit has the (*S*) absolute configuration, which was confirmed (Fig. 6 and Figs. S2 and S3 for inflorescence results).

**Fig. 7:**
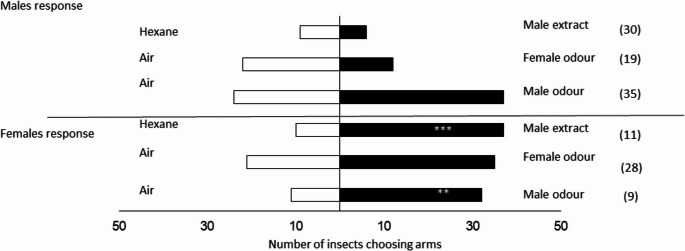
Behavioural response of *S. mangiferae* males and females from a Seropédica (RJ) population in the Y-tube olfactometer to volatiles from live conspecific males and females, as well as to male aeration extracts. The number in parentheses indicates non-responsive insects. * Significant differences between treatment and control (ꭓ^2^, *P* < 0.05)

**Fig. 8:**
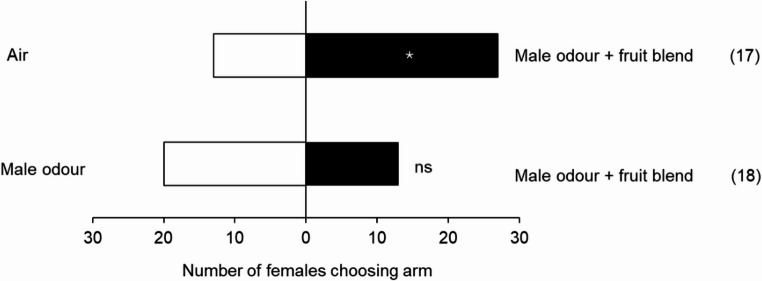
Behavioural response of *S. mangiferae* females from a Seropédica (RJ) population in the Y-tube olfactometer to volatiles from live conspecific males and odour from fruits (var. Coquinho) and inflorescence (var. Tommy). The number in parentheses indicates the non-responsive insects. * Significant differences between treatment and control (ꭓ^2^, *P* < 0.05)

Similar results were observed when analysing the behaviour of *S. mangiferae* males and females from a Seropédica (RJ) population. Females showed a preference for live male odours (χ²= 10.256, *P* = 0.001) and for male aeration extracts (χ²= 15.510, *P* ≤ 0.001). The preference for female odours was marginal when contrasted with air (χ²= 3.500, *P* = 0.06) (Fig. 7; Table S2). Males did not show a preference for any of the odours evaluated (Fig. 7; Table S2). When male odour was combined with fruit and inflorescence odour, females preferred the combined odours compared to air (χ²= 4.9, *P* = 0.027), but when male odour + fruit and inflorescence odour was contrasted with male odour, females did not show a preference for either (χ²= 1.484, *P* = 0.223) (Fig. 8).

### Male and Female Volatile Profiles

The chemical analysis of male and female extracts revealed highly similar volatile profiles (Fig. S4, Table S3), with no significant qualitative differences. Several compounds identified in the insects’ volatile profiles, such as β-pinene, myrcene and (*E*)-ocimene, were also present in mango tree extracts (Figs. S1, S2, and S4), along with α-copaene, β-caryophyllene, α-humulene and methyl salicylate. Additionally, the total amount of volatiles released by the insects over six days showed a decreasing trend (Fig. S5). A second set of insects, from which volatiles were collected at 48-hour intervals, confirmed a marked decrease in volatile emissions on the second day (Table S4). Again, no sex-specific or insect-specific compounds could be identified, and a high variability in volatile emission was observed among replicates.

## Discussion

Our results confirm the importance of plant kairomones in the chemical communication of *S. mangiferae*, aligning with findings for other curculionids (Piñero and Prokopy 2003; Tinzaara et al. 2007; Guarino et al. 2011; Magalhães et al. 2016). Interestingly, only *S. mangiferae* females exhibited a preference for these kairomones, consistently observed in two separate populations evaluated in different laboratories. In addition, only *S. mangiferae* females can discriminate between males and females through volatiles, moving toward the odour of live males and male aeration extracts. Males were not attracted to these conspecific volatiles of both sexes, and the females did not respond to the odour of other females, supporting the hypothesis that these compounds are only released by males and act as attractants exclusively for females. The chemical analysis of volatile samples containing the volatiles emitted by *S. mangiferae* did not identify male-specific compounds that could explain this attraction. It is possible that the male-specific compounds are produced in trace amounts, below the detection limits of the equipment used. While the behavioural assays strongly suggest a role in sexual attraction, the precise function as a sex pheromone remains to be confirmed until the active compounds are chemically identified and the behavioural role of the synthetic pheromone is evaluated. Another interesting result is that females were not attracted to male odour during the photophase, and they showed a significant preference for the arm of the olfactometer with air. This result suggests that maybe males were producing an alarm or defensive compound. The bioassay results using insect-derived volatiles suggest a possible nocturnal component in the reproductive behaviour of *Sternochetus mangiferae*. Nocturnal activity in this species was originally reported by Subramanyam (1926, cited in De Graaf 2010). However, a more recent study with a Brazilian population did not detect differences in reproductive behaviour between photophase and scotophase (Catafesta et al. 2023). The absence of diel differences reported by Catafesta et al. (2023) may be related to the experimental conditions, as those assays were conducted in artificial arenas that allowed proximity and visual contact between individuals. Olfactometer assays, on the other hand, limit interactions to chemical volatile cues only, requiring insects to rely exclusively on olfactory signals. From an ecological perspective, nocturnal chemical signalling may reduce exposure to visually oriented predators and parasitoids, thereby providing a safer context for intraspecific communication; this, however, would require that natural enemies exert a high enough selection pressure to cause a life history shift in evolutionary terms. Consequently, odour-mediated interactions in *S. mangiferae* may be more effectively expressed under nocturnal conditions. Further studies under controlled diel conditions are needed to strengthen and validate the observations reported here. The observed female preference for plant kairomones may be linked to oviposition needs, as females are likely more responsive to plant volatiles to locate suitable sites for egg-laying on mango fruits. Additionally, while numerous studies have demonstrated a synergistic effect of pheromones and plant kairomones in Curculionidae species (Giblin-Davis et al. 1996; Piñero and Prokopy 2003; Guarino et al. 2011; Magalhães et al. 2016; Vacas et al. 2017), our study did not observe this effect. The lack of male response to plant volatiles and the absence of a synergistic effect between conspecific odours and mango volatiles may be related to several factors, like the perennial nature of mango trees, which are not frequently relocated or replanted, unlike annual or semi-perennial crops such as cotton, palm, or plum. In such systems, host-location cues may be less critical for males once they are established within the orchard. In addition, it is possible that males do not respond to plant kairomones during the scotophase. In the literature, several Curculionidae species exhibit peaks in reproductive activity and pheromone release during the photophase, such as *Anthonomus grandis* and *Anthonomus eugenii* (Gueldner and Wiygul 1978; Eller and Palmquist 2014). It can therefore be hypothesized that, during the scotophase, males of *S. mangiferae* may prioritize pheromone production for female attraction and consequently show reduced responsiveness to plant kairomones. *Sternochetus mangiferae* typically moves only short distances within orchards (De Graaf 2010), suggesting that host plant volatiles may function primarily as cues for assessing phenological changes in mango trees rather than for long-range host location. Females were attracted to the synthetic blend containing (*S*)-linalool. This result aligns with the findings from chiral analysis of volatile extracts from mango inflorescences and fruits, which confirmed the presence of only the (*S*) enantiomer. The absolute configuration of semiochemicals plays a critical role in the biological responses of insects (Sims et al. 2022). In general, insect olfactory systems can distinguish between enantiomers, as demonstrated in this study for *S. mangiferae* and in previous studies for *Alphitobius diaperinus*,* A. grandis* and several other species (Hassemer et al. 2016; Magalhães et al. 2018; Sims et al. 2022). Further studies could investigate whether virgin *S. mangiferae* individuals of varying ages display preferences like those observed here. In addition, as shown in other studies, kairomone blends may exhibit redundancy among components. Therefore, experiments in which individual compounds are sequentially removed could be conducted to evaluate whether all components are necessary for eliciting a behavioural response. Although insects are generally more strongly attracted to blends of volatiles than to one or two individual compounds, it remains necessary to determine, for *S. mangiferae*, whether all components proposed here are required or whether a simplified blend could be equally effective. It is noteworthy that a high proportion of non-responsive insects was observed across all bioassays. This pattern was particularly evident in assays using insect-derived odours from volatile extracts or live insects, in which non-response rates were generally high. However, when females were exposed to male odours, the proportion of non-responders was consistently lower. For females from Ghana, whose physiological status was unknown, approximately 25 individuals did not respond when exposed to male odour during the scotophase. In the Brazilian population, the number of non-responsive females was even lower (11 when exposed to male volatile extracts and 9 when exposed to live males as the odour source). When plant volatiles were tested, the number of non-responsive insects was also high, but generally lower than that observed in assays using insect volatiles. Overall, this pattern suggests that the high number of non-responders in certain treatments reflects limited behavioural activation by specific odour sources rather than methodological inconsistency. Although this study clearly demonstrates that females respond to odours released by conspecific males, it remains unclear whether this response is driven solely by male-produced volatiles or by a combination of cues, including male odours, vibrational (stridulatory) (Catafesta et al. 2023) signals, and mango volatiles associated with insects carrying plant residues. These aspects, together with the identification of a potential aggregation pheromone in this species, require further investigation. Volatile profile analysis revealed chemical similarities between compounds emitted by *S. mangiferae* and those from mango, suggesting potential contamination from insect-fruit interactions. We hypothesize that such contamination explains the presence of these compounds in insect headspace extracts, as their quantities decreased over days of aeration. Nevertheless, *S. mangiferae* may exploit these compounds as cues, contaminating themselves and becoming emitters of mango-derived compounds. Since both male and female insects produce qualitatively similar profiles, and response to live female odour was not observed in olfactometry bioassays, it is possible that minor, undetected compounds contribute to female behavioural preference for male odour. These findings indicate that mango volatiles play a crucial role in female host plant location, and this apparent contamination may serve as a chemical cue facilitating encounters and mating. Future studies should further investigate male-specific minor compounds that may complement attraction mechanisms among individuals. This study provides the first evidence that chemical cues from both males and mango trees play a central role in the ecology of *S. mangiferae*. We demonstrated that volatiles from mango fruits and inflorescences, particularly blends containing (*S*)-linalool, elicit strong behavioural responses in females of both Ghanaian and Brazilian populations. Females also responded to live male odours, indicating that, in addition to kairomones, conspecific volatiles contribute to female attraction. As observed for other Curculionidae, host plant chemicals are used by *S. mangiferae*, suggesting that future monitoring strategies for this species will likely require a combination of host kairomones and sex- or aggregation pheromones. Moreover, further studies should investigate the influence of age and mating status on pheromone production and on behavioural responses to both host-derived kairomones and conspecific signals.

## Supplementary Information

Below is the link to the electronic supplementary material.

Supplementary Material 1 (DOCX 292 KB)

## Data Availability

No datasets were generated or analysed during the current study.
