# Supplementary material for: Influence of Conspecific Male Odour and Host Kairomones on the Behaviour of Sternochetus mangiferae, a Pest of Mangoes in Brazil and Ghana
Source: J Chem Ecol. 2026 Mar 17;52(2):29. doi: 10.1007/s10886-026-01696-5 (PMC12995999; doi:10.1007/s10886-026-01696-5)
Supplement: Supplementary file 1 — Supplementary Material 1 (DOCX 292 KB) [file 10886_2026_1696_MOESM1_ESM.docx]

Supplementary material manga weevil paper

**Table S1.** Mean amount ± error standard (µg/hour) of volatiles identified from fruits (var Coquinho) (n = 5) and inflorescences (var Tommy) (n = 3). RI calculated using a DB-5 MS column (30m x 0.25mm. 0.25mm).

| # | Compounds | RI^a^ | Fruit Coquinho | Inflorescence Tommy |  |  |
| --- | --- | --- | --- | --- | --- | --- |
| 1 | Methyl-2-methyl butyrate^*^ | 780 | - | 0.186 ± 0..078 |  |  |
| 2 | (*E*)-2-hexen-1-ol | 861 | - | 0.09 ± 0.017 |  |  |
| 3 | γ-Butyrolactone | 920 | 0.035± 0.009 | 0.044 ± 0.027 |  |  |
| 4 | Furfural^*^ | 930 | - | 0.067 ± 0.023 |  |  |
| 5 | Benzaldehyde | 961 | 0.066±0.018 | 0.194 ± 0.014 |  |  |
| 6 | β-pinene | 978 | Traces | 1.288 ± 0.588 |  |  |
| 7 | 6-Methyl-5-hepten-2-one | 985 | 0.053 ± 0.011 | 1.000 ± 0.298 |  |  |
| 8 | Myrcene | 991 | 3.473 ± 1.682 | 15.693 ± 7.458 |  |  |
| 9 | 3-Carene | 1016 | 7.404 ± 5.368 | 51.716 ± 18.459 |  |  |
| 10 | Limonene | 1031 | 0.131 ± 0.131 | 1.599 ± 0.922 |  |  |
| 11 | (*Z*)-Ocimene | 1037 | 0.927 ± 0.927 | 14.381± 6.139 |  |  |
| 12 | (*E*)-Ocimene | 1050 | 0.293 ± 0.270 | 6.537 ± 2.844 |  |  |
| 13 | 2-Methoxyphenol | 1086 | 0.229 ± 0195 | 1.523 ± 0.749 |  | 0.229 ± 0195 |
| 14 | α-Terpinolene* | 1091 | 0.562 ± 0.543 | 16.483 ± 8.104 |  |  |
| 15 | *trans*-Linalool oxide | 1095 | 0.051 ± 0.046 | 0.614± 0.482 |  |  |
| 16 | Methyl benzoate | 1099 | 1.435 ± 1.406 | 23.126 ± 14.514 |  |  |
| 17 | (*S*)-Linalool | 1106 | 0.127 ± 0.037 | 0.549 ± 0.157 |  |  |
| 18 | (*E*)-4.8-Dimethyl-1.3.7-nonatriene | 1115 | 0.337 ± 0.337 | 13.362 ± 8.174 |  |  |
| 19 | Allocimene* | 1120 | 0.306 ± 0.256 | 0.865 ± 0.925 |  |  |
| 20 | Ethyl benzoate* | 1176 | 4.655 ± 4.214 | 54.1596 ± 21.222 |  |  |
| 21 | Methyl salicylate | 1193 | 0.625 ± 0.149 | 0.706 ± 0.716 |  |  |
| 22 | Dodecane | 1200 | 3.648 ± 3.521 | 0.119 ± 0.066 |  |  |
| 23 | Methyl-2-methoxybenzoate | 1212 | 0.017 ± 0.011 | 0.264 ± 0.072 |  |  |
| 24 | α-Copaene | 1389 | - | 7.678 ± 6.034 |  |  |
| 25 | α-Gurjunene*^*^ | 1424 | 0.228 ± 0.093 | 48.818 ± 19.758 |  |  |
| 26 | (*E*)-Caryophillene | 1439 | 1.870 ± 1.693 | 6.728 ± 2.463 |  |  |
| 27 | α-Humulene | 1475 | 0.863 ± 0.816 | 3.844 ± 1.266 |  |  |
| 28 | Sesquiterpeno unknonw | 1479 | - | 4.884 ± 1.669 |  |  |
| 29 | (*R or S*)-Germacrene D* | 1480 | 0.213 ± 0.189 | 6.242 ± 2.235 |  |  |
| 30 | β-Selinene* | 1489 | 0.566 ± 0.280 | 8.728 ± 2.840 |  |  |
| 31 | (*E.E*)-α-Farnesene | 1507 | 8.381 ± 5.542 | 41.078 ± 18.154 |  |  |
| 32 | δ-Cadinene | 1532 | - | 3.754 ± 2.236 |  |  |

* compounds tentatively identified based on retention index and comparison of fragmentation patterns with data from the NIST library.

^a^ Retention index

**Table S2.** Results of the chi-square statistical test for behavioural assays.

| Figure 2- Females from Kumasi- Ghana responding to the odour of conspecifics | | |
| --- | --- | --- |
|  | Time | |
|  | Day | Night |
| Male odour vs Air | χ2 = 5.786, p = 0.016 | χ2 = 7.246, p = 0.007 |
| Female odour vs Male odour | - | χ2 = 0.209, p = 0.647 |
| Female odour vs Air | - | χ2 = 5.000, p = 0.025 |
| Figure 3 - Males from Kumasi- Ghana responding to the odour of conspecifics - | | |
|  | Day | Night |
| Male odour vs Air | - | χ2 = 1.884, p = 0.169 |
| Female odour vs Male odour | - | χ2 = 0.000, p = 1.000 |
| Female odour vs Air | χ2 = 2.373, p = 0.124 | χ2 = 0.000, p = 1.000 |

Figure 4. Response of males and females from Kumasi- Ghana in a linear olfactometer to the odour of mango VOCs from a synthetic solution.

| Male response | Night |  |
| --- | --- | --- |
| Synthetic solution w/ R-linalool vs Hexane | χ2 = 0.529, p = 0.466 | |
| Synthetic solution w/ S-linalool vs Hexane | χ2 = 0.473, p = 0.491 | |
| Synthetic solution w/ linalool vs Hexane | χ2 = 0.428, p = 0.513 | |
| Female response | Night | |
| Synthetic solution w/ R-linalool vs Hexane | χ2 = 1.147, p = 0.225 | |
| Synthetic solution w/ S-linalool vs Hexane | χ2 = 3.483, p = 0.050 | |
| Synthetic solution w/ linalool vs Hexane | χ2 = 0.800, p = 0.371 | |

Figure 5. Response of males and females from Seropedica - Brazil in a Y olfactometer to the odour of mango VOCs from a synthetic solution.

| Male response | Night |
| --- | --- |
| Synthetic solution w/ S-linalool vs Hexane | χ2 = 0.290, p = 0.590 |
| Female response | Night |
| Synthetic solution w/ S-linalool vs Hexane | χ2 = 4.172, p = 0.041 |

| Figure 7- Males and Females from Seropédica - Brazil responding to the odour of conspecifics |
| --- |

| Male response | Night |
| --- | --- |
| Male extract vs Hexane | χ2 = 0.600, p = 0.439 |
| Female odour vs Air | χ2 = 2.941, p = 0.080 |
| Male odour vs Air | χ2 = 2.771, p = 0.096 |
| Female response | Night |
| Male extract vs Hexane | χ2 = 15.511, p ≤ 0.001 |
| Female odour vs Air | χ2 = 3.500 , p = 0.061 |
| Male odour vs Air | χ2 = 10.256, p = 0.001 |

**Table S3.** Average quantity of volatiles (ng/insect/day ± standard error) released by *S. mangiferae* females and males in aeration extracts. Retention index on DB-5MS column.

| Compounds |  |  | *S. mangiferae* females |  | *S. mangiferae* males |
| --- | --- | --- | --- | --- | --- |
|  | RI^a^ literature^b^ | RI^a^ |  |  |  |
|  |  |  |  |  |  |
| β-Thujene* | 931 | 925 | 1.284 ± 0.358 |  | 0.399 ± 0.066 |
| α-Pinene | 939 | 929 | 3.223 ± 0.716 |  | 2.414 ± 0.885 |
| Camphene | 953 | 944 | 3.428 ± 1.494 |  | 2.252 ± 1.066 |
| Benzaldehyde | 961 | 957 | 5.392 ±1.926 |  | 3.606 ± 2.232 |
| β-Pinene | 980 | 971 | 1.809 ±0.376 |  | 0.906 ± 0.378 |
| Unknown 1 |  | 975 | 3.100 ±0.713 |  | 1.658 ± 0.297 |
| Myrcene | 991 | 988 | 25.111 ±7.899 |  | 2.565 ± 1.301 |
| Decane | 1000 | 1000 | 1.664 ±0.356 |  | 0.491 ± 0.262 |
| Octanal | 1004 | 1005 | 5.229 ±1.260 |  | 2.623 ±1.101 |
| 2-Ethylhexanol | 1029 | 1026 | 2.599 ±1.266 |  | 0.156 ± 0.069 |
| Benzylalcohol | 1033 | 1032 | 1.529 ±0.427 |  | 0.352 ± 0.192 |
| (*E*)-Ocimene | 1050 | 1045 | 13.956± 6.608 |  | 9.932 ± 5.242 |
| Unknown 2 |  | 1068 | 0.986±0.369 |  | 0.904 ± 0.466 |
| (*E*)-2-Nonenal^*^ | 1062 | 1075 | 3.365±0.947 |  | 1.388 ± 0.658 |
| Undecane | 1100 | 1100 | 2.011±0.439 |  | 0.944 ± 0.345 |
| Nonanal | 1005 | 1107 | 14.390±5.650 |  | 6.889 ± 3.237 |
| Phenethylalcohol | 1113 | 1108 | 1.507±1.641 |  | 0.000 ± 0.000 |
| Unknown 3 |  | 1112 | 0.961±0.614 |  | 0.866 ± 0.304 |
| Unknown4 |  | 1136 | 1.136±1.209 |  | 1.248 ± 0.502 |
| Menthone* | 1154 | 1154 | 1.669±0.88 |  | 1.181 ± 0.792 |
| (*E*)-2-Dodecen-1-ol* |  | 1166 | 0.516±0.121 |  | 0.221 ± 0.108 |
| p-Menthan-1-ol* |  | 1176 | 3.573±1.881 |  | 0.977 ± 0.434 |
| Methyl salicylate | 1190 | 1187 | 1.052±0.255 |  | 0.487 ± 0.221 |
| α-Terpineol | 1189 | 1192 | 1.372±0.498 |  | 0.464 ± 0.403 |
| Dodecane | 1200 | 1200 | 8.448±2.594 |  | 5.221 ± 2.102 |
| Decanal | 1005 | 1206 | 3.651±1.275 |  | 1.982 ± 0.801 |
| 2-Phenoxyethanol* | 1226 | 1217 | 2.587±1.725 |  | 0.422 ± 0.132 |
| Benzothiazole | 1221 | 1222 | 1.235±0.502 |  | 0.515 ± 0.155 |
| (*Z*)-3-Hexenyl valerate | 1236 | 1232 | 0.790±0.360 |  | 0.320 ± 0.099 |
| 1-Phenoxypropan-2-ol* | 1246 | 1241 | 0.464±0.161 |  | 0.240 ± 0.102 |
| Linalyl acetate* | 1257 | 1252 | 0.793±0.267 |  | 0.528 ± 0.208 |
| Nonanoic acid | 1280 | 1266 | 2.185±0.807 |  | 1.014 ± 0.527 |
| Tridecane | 1300 | 1300 | 5.183±3.169 |  | 1.538 ± 0.754 |
| Undecanal | 1305 | 1303 | 1.081±0.801 |  | 2.866 ± 1.121 |
| Decanoic acid | 1380 | 1363 | 4.347±2.574 |  | 1.053 ± 0.553 |
| α-Copaene* | 1377 | 1367 | 11.094±6.750 |  | 7.458 ± 2.991 |
| Tetradecane | 1400 | 1400 | 5.659±2.864 |  | 2.672 ± 0.974 |
| α-Gurjunene* | 1409 | 1407 | 28.915±17.160 |  | 28.447 ± 10.267 |
| Dodecanal | 1405 | 1413 | 3.456±1.992 |  | 3.340 ± 1.239 |
| (*E*)-Caryophyllene | 1418 | 1421 | 3.459±1.372 |  | 2.212 ± 0.818 |
| Geranylacetone | 1453 | 1448 | 2.170±0.992 |  | 1.091 ± 0.329 |
| α-Humulene | 1454 | 1459 | 1.485±0.653 |  | 0.640 ± 0.304 |
| β-Selinene* | 1492 | 1489 | 2.724±0.835 |  | 1.126 ± 0.694 |

* compounds tentatively identified based on retention index and comparison of fragmentation patterns with data from the NIST library.

^a^ Retention index

^b^ references for the RI literature can be find on the reference list at the end of this document

**Table S4.** Average quantity of volatiles (ng/insect/day ± standard error) released by 35 *S. mangiferae* females (n=5) and males (n=5) in aeration extracts. Retention index on DB-5MS column.

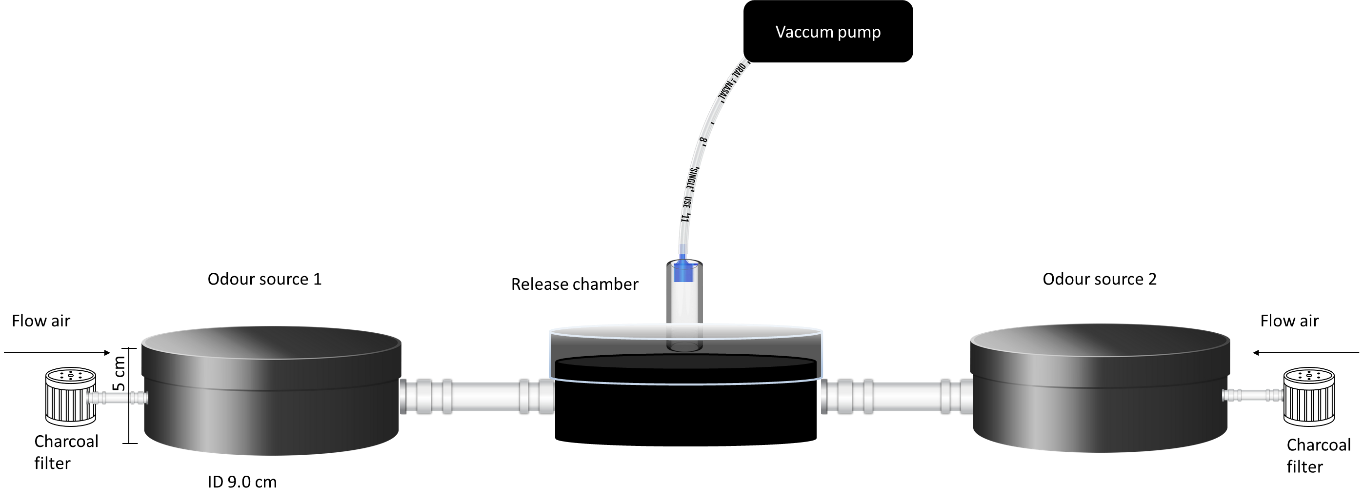


**Figure S1.** Linear olfactometer used in the bioassays. The chamber has an internal diameter of 9.0 cm and a height of 5.0 cm. The lid of the central chamber was transparent, while the other chambers were covered with black tape. Flow of 0.6 l/min


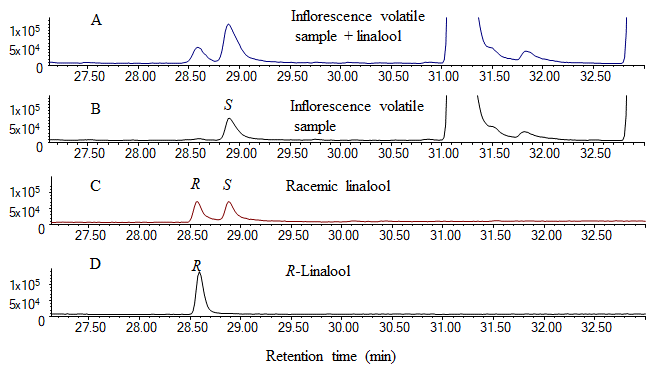


**Figure S2.** Total ion chromatograms (TIC) of racemic linalool, (S)-linalool, and volatile extracts from mango inflorescence and fruit samples. Column: β- DEX-360.


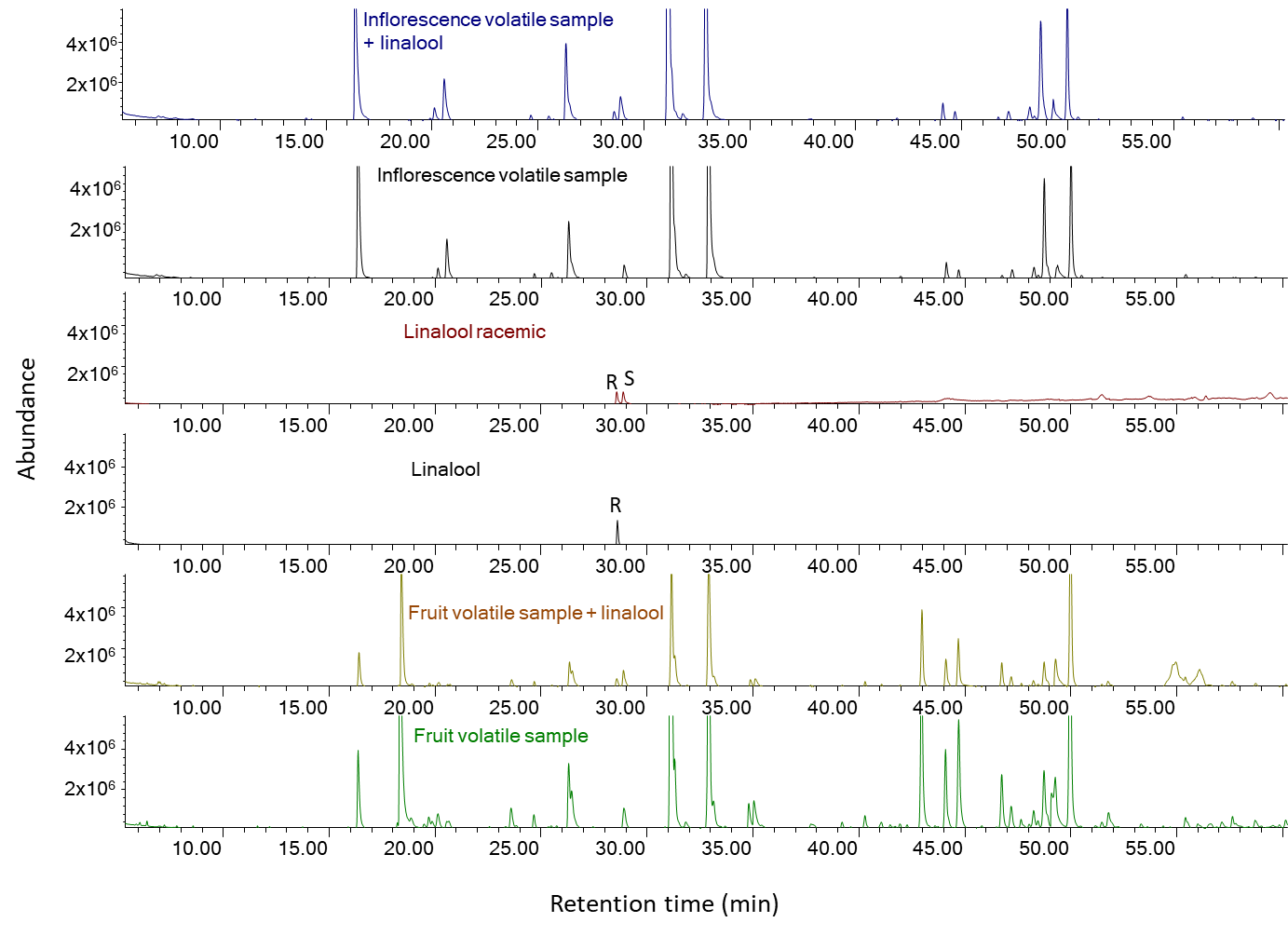


**Figure S3**. A. GC co-injection of a mango inflorescence (var. Tommy) aeration extract with racemic linalool, B. inflorescence sample aeration extract, C. synthetic racemic linalool, D. synthetic (*R*)-linalool. Column: β- DEX-360.

**
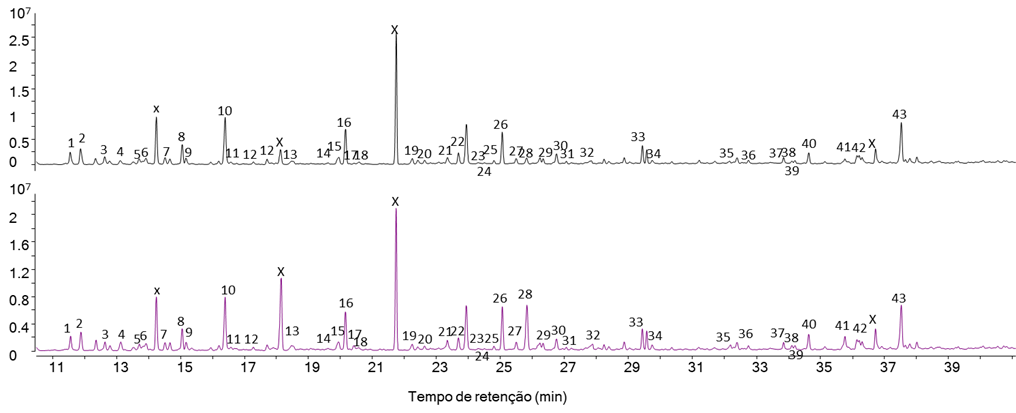
** **Figure S4.** GC-MS analysis of aeration extracts of A. male and B. female *S. mangiferae*. 1) β-thujene, 2) α-pinene, 3) camphene, 4) benzaldehyde, 5) (+)-3-carene, 6) β-pinene, 7) unknown, 8) decane, 9) octanal, 10) 2-ethylhexan-1-ol, 11) benzyl alcohol, 12) 3-carene, 13) unknown, 14) (*E*)-2-nonenal, 15) undecane, 16) nonanal, 17) phenylethyl alcohol, 18) unknown, 19) 4-methylpentyl 2-methylpropanoate, 20) menthone, 21) (*E*)-2-dodecenol, 22) p-menthan-1-ol, 23) methyl salicylate, 24) α-terpineol, 25) dodecane, 26) decanal, 27) 2-phenoxyethanol, 28) benzothiazole, 29) (*Z*)-3-hexenyl isovalerate, 30) 1-phenoxy-2-propanol, 31) linalyl acetate, 32) nonanoic acid, 33) tridecane, 34) undecanal, 35) decanoic acid, 36) α-copaene, 37) tetradecane, 38) α-gurjunene, 39) dodecanal, 40) (*E*)-caryophyllene, 41) geranylacetone, 42) α-humulene, 43) β-selinene.

B

A


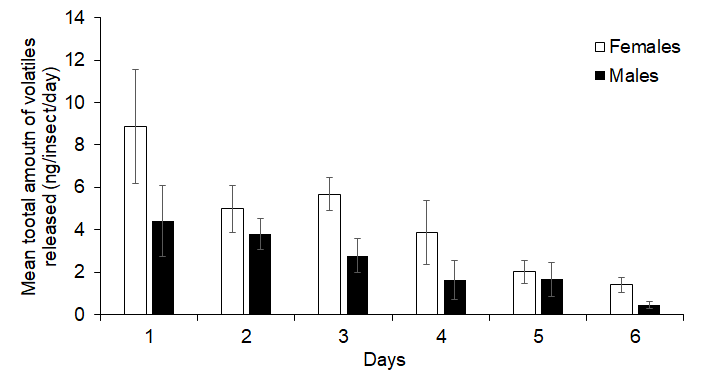


**Figure S5.** Mean total amount of volatiles (ng/insect/day ± SE) released by males and females over six consecutive days.

References retention index

**Camphene, β-thujene, α-pinene, benzaldehyde, b-pinene, myrcene, menthone, methyl salicylate, a-terpineol, linaly acetate, nonanoic acid, decanoic acid, β-caryophyllene, copaene, α-gurjunene, humulene, genranyl acetone**

Adams, R.P. 1995. Identification of essential oil components by gas chromatography/mass spectrometry. Allured Publishing Corporation, Carol Stream, IL.

**1-Phenoxypropan-2-ol**

[Andriamaharavo, N.R.](https://webbook.nist.gov/cgi/cbook.cgi?Author=Andriamaharavo%2C+N.R.&Mask=2000), **Retention Data. NIST Mass Spectrometry Data Center.**, NIST Mass Spectrometry Data Center, 2014. [[all data](https://webbook.nist.gov/cgi/cbook.cgi?Source=2014AND%2319410M&Mask=2000)]

**Benzyl achool, 2-nonenal, (E)-**

[Pino, J.A.](https://webbook.nist.gov/cgi/cbook.cgi?Author=Pino%2C+J.A.&Mask=2000); [Marquez, E.](https://webbook.nist.gov/cgi/cbook.cgi?Author=Marquez%2C+E.&Mask=2000); [Quijano, C.E.](https://webbook.nist.gov/cgi/cbook.cgi?Author=Quijano%2C+C.E.&Mask=2000); [Castro, D.](https://webbook.nist.gov/cgi/cbook.cgi?Author=Castro%2C+D.&Mask=2000), *Volatile compounds in noni (Morinda citrifolia L.) at two ripening stages*, **Ciencia e Technologia de Alimentos**, 2010, 30, 1, 183-187, [https://doi.org/10.1590/S0101-20612010000100028](https://dx.doi.org/10.1590/S0101-20612010000100028) . [[all data](https://webbook.nist.gov/cgi/cbook.cgi?Source=2010PIN%2FMAR183-187&Mask=2000)]

**Phenethyl alcohol**

**Leffingwell and Alford, 2011**
[Leffingwell, J.](https://webbook.nist.gov/cgi/cbook.cgi?Author=Leffingwell%2C+J.&Mask=2000); [Alford, E.D.](https://webbook.nist.gov/cgi/cbook.cgi?Author=Alford%2C+E.D.&Mask=2000), *Volatile constituents of the giant pufball mushroom (Calvatia gigantea)*, **Leffingwell Rep.**, 2011, 4, 1-17. [[all data](https://webbook.nist.gov/cgi/cbook.cgi?Source=2011LEF%2FALF1-17&Mask=2000)]

**Benzothiazole:**

**Pino, Marbot, et al., 2005**
[Pino, J.A.](https://webbook.nist.gov/cgi/cbook.cgi?Author=Pino%2C+J.A.&Mask=2000); [Marbot, R.](https://webbook.nist.gov/cgi/cbook.cgi?Author=Marbot%2C+R.&Mask=2000); [Rosado, A.](https://webbook.nist.gov/cgi/cbook.cgi?Author=Rosado%2C+A.&Mask=2000); [Vázquez, C.](https://webbook.nist.gov/cgi/cbook.cgi?Author=Vazquez%2C+C.&Mask=2000), *Volatile constituents of genipap (Genipa americana L.) fruit from Cuba*, **Flavour Fragr. J.**, 2005, 20, 6, 583-586, [https://doi.org/10.1002/ffj.1491](https://dx.doi.org/10.1002/ffj.1491) . [[all data](https://webbook.nist.gov/cgi/cbook.cgi?Source=2005PIN%2FMAR583-586&Mask=2000)]

**z-3-Hexenyl valerate**

[Zhao C.X.](https://webbook.nist.gov/cgi/cbook.cgi?Author=Zhao+C.X.&Mask=2000); [Li, X.N.](https://webbook.nist.gov/cgi/cbook.cgi?Author=Li%2C+X.N.&Mask=2000); [Liang Y.Z.](https://webbook.nist.gov/cgi/cbook.cgi?Author=Liang+Y.Z.&Mask=2000); [Fang H.Z.](https://webbook.nist.gov/cgi/cbook.cgi?Author=Fang+H.Z.&Mask=2000); [Huang L.F.](https://webbook.nist.gov/cgi/cbook.cgi?Author=Huang+L.F.&Mask=2000); [Guo F.Q.](https://webbook.nist.gov/cgi/cbook.cgi?Author=Guo+F.Q.&Mask=2000), *Comparative analysis of chemical components of essential oils from different samples of Rhododendron with the help of chemometrics methods*, **Chemom. Intell. Lab. Syst.**, 2006, 82, 1-2, 218-228, [https://doi.org/10.1016/j.chemolab.2005.08.008](https://dx.doi.org/10.1016/j.chemolab.2005.08.008) . [[all data](https://webbook.nist.gov/cgi/cbook.cgi?Source=2006ZHA%2FLI218-228&Mask=2000)]

**β-Selinene**

De Kraker, J.-W., Schurink, M., Franssen, M.C.R., K�nig, W.A., de Groot, A., and Bouwmeester, H.J. 2003. Hydroxylation of sesquiterpenes by enzymes from chicory (Cichorium intybus L.) roots. Tetrahedron. 59:409-418.
